# Tizoxanide Promotes Apoptosis in Glioblastoma by Inhibiting CDK1 Activity

**DOI:** 10.3389/fphar.2022.895573

**Published:** 2022-05-25

**Authors:** Si Huang, Jingxian Xiao, Junyong Wu, Jiayi Liu, Xueping Feng, Chengdong Yang, Daxiong Xiang, Shilin Luo

**Affiliations:** ^1^ Department of Pharmacy, The Second Xiangya Hospital, Central South University, Changsha, China; ^2^ Hunan Provincial Engineering Research Centre of Translational Medicine and Innovative Drug, Changsha, China; ^3^ School of Medical Science, Hunan University of Medicine, Huaihua, China; ^4^ Department of Radiology, The Second Xiangya Hospital, Central South University, Changsha, China; ^5^ Institute of Medical Sciences, Xiangya Hospital, Central South University, Changsha, China; ^6^ Department of Psychiatry, The Second Xiangya Hospital, Central South University, Changsha, China

**Keywords:** tizoxanide, glioblastoma, Cdk1, cell cycle, apoptosis

## Abstract

The antiparasitic drug nitazoxanide (NTZ) has received considerable attention for its potential in cancer therapy. In this study, we demonstrate that tizoxanide (TIZ), an active metabolite of NTZ, exhibits antiglioma activity *in vitro* and *in vivo* by inducing G2/M cell cycle arrest and apoptosis. *In vitro*, TIZ dose-dependently inhibited the proliferation of U87, U118, and A172 human glioblastoma (GBM) cells at 48 h with IC_50_ values of 1.10, 2.31, and 0.73 µM, respectively. Treatment with TIZ (1 and 10 µM) also dose-dependently inhibited the colony formation of these GBM cells and accumulated ROS damage in the nucleus. In silico target fishing combined with network pharmacological disease spectrum analyses of GBM revealed that cycle-dependent kinase 1 (CDK1) is the most compatible target for TIZ and molecular docking by Molecule Operating Environment (MOE) software confirmed it. Mechanistically, TIZ inhibited the phosphorylation of CDK1 at Thr161 and decreased the activity of the CDK1/cyclin B1 complex, arresting the cell cycle at the G2/M phase. TIZ may induce apoptosis *via* the ROS-mediated apoptotic pathway. *In vivo*, TIZ suppressed the growth of established subcutaneous and intracranial orthotopic xenograft models of GBM without causing obvious side effects and prolonged the survival of nude mice bearing glioma. Taken together, our results demonstrated that TIZ might be a promising chemotherapy drug in the treatment of GBM.

## Introduction

Glioblastoma multiforme (GBM) is one of the most aggressive and malignant human brain tumors. Although progress has been made concerning its standard therapeutic regimen, which includes radiation therapy and temozolomide chemotherapy following surgical resection over the years, the current patient’s overall median survival is approximately 15 months, and the 5-year survival rate is 4%–5% (Batash et al., 2017). Temozolomide (TMZ) is the oral first-line drug for the treatment of GBM in the clinic, and it increases the survival term by approximately 3 months after combination with radiation (Johnson et al., 2014). However, at least 50% of TMZ-treated patients do not respond to TMZ, and side effects and acquired resistance limit the effective application of TMZ (Lee, 2016). Therefore, more research focusing on discovering new therapeutic drugs for GBM is urgently needed.

Tizoxanide (TIZ), a nitrothiazolamide compound, is a rapid *in vivo* active metabolite of nitazoxanide (NTZ) that was approved by the US Food and Drug Administration (FDA) as an antiparasitic drug. TIZ has shown a broad spectrum of pharmacological functions in extracellular and intracellular protozoans, anaerobic and microaerophilic bacteria, helminths, and viruses (Anderson and Curran, 2007; Muller and Hemphill, 2011; Rossignol, 2014). As in-depth studies are developed, NTZ has been reported to express inhibitory effects by intervening in the crucial metabolic and pro-death signalings in tumor cells, such as autophagy, detoxifying enzyme overexpression, anti-cytokine activity, and c-Myc inhibition (Di Santo and Ehrisman, 2014; Shakya et al., 2018). A study has shown that NTZ can inhibit late-stage autophagy and promote the ING1-induced cell cycle arrest in GBM (Wang et al., 2018). However, the direct binding targets and the underlying mechanisms of the metabolite TIZ in anti-glioblastoma are still unclear.

As part of a research project on drug repurposing, in the present study, we found that treating U87, U118, and A172 glioblastoma cells with TIZ inhibited cell proliferation and colony formation. Using a strategy based on network pharmacological disease target analysis and computerized compound-target seeking, we focused on the cyclin-dependent kinase (CDK) family, especially CDK1, as a target of TIZ for its anti-GBM function. The molecular docking of TIZ with CDK1 revealed that TIZ blocked the active sites with high affinity. Furthermore, flow cytometry and immunoblotting analysis results indicated that TIZ induced apoptosis in U87MG cells by arresting the cell cycle at G2/M phases through the suppression of the CDK1/Cyclin B1 complex. TIZ also blocked the propagation of GBM and prolonged the survival rate in mice bearing orthotopic tumors. Taken together, our findings demonstrate the potential inhibitory efficacy of TIZ for GBM *in vitro* and *in vivo*, and CDK1 activity inhibition-induced TIZ-associated G2/M cell cycle arrest is the underlying molecular mechanism.

## Materials and Methods

### Antibodies and Reagents

Antibodies to the following targets were used: anti-p-CDK1 Thr161 (BBI Sci, #D155339), anti-CDK1 (BBI Sci, #D260158), catalog number #8516S for anti-p-Rb Ser807/811 (Cell Signaling Technology, #), anti-Rb (Santa Cruz Biotechnology, #sc-74563), anti-CCNA1 (BBI Sci, #D220507), anti-CCNB1 (BBI Sci, #D160234), anti-GAPDH (Santa Cruz Biotechnology, #sc-47724), Annexin V-PE/7AAD Kit (Solarbio Science & Technology, #CA1030), Reactive Oxygen Species assay Kit (US Everbright INC, #R6033), and Cremophor EL (Sigma, #C5135). Tizoxanide (#S83937), N-acetyl-L-cysteine (#S20137), and D-luciferin sodium salt (#S19261) were purchased from Yuanye Bio-Technology Co. (Shanghai, China). Tizoxanide was dissolved in dimethyl sulfoxide (DMSO) at a stock concentration of 10 mM and diluted with the relevant medium for the *in vitro* experiments. The final concentration of DMSO was less than 0.1%. For *in vivo* studies, tizoxanide was dissolved in Cremophor EL/ethanol (50:50) to ensure solubility and then diluted in ultrapure water.

### Cell Culture

The human glioblastoma cell line U87MG was purchased from the Cell Resource Center at the Institute of Basic Medical Sciences, Chinese Academy of Medical Sciences (Beijing, China); U87MG-luc was obtained from Shanghai Model Organisms Center, Inc., (Shanghai, China); U118 MG was acquired from Procell Life Science & Technology Co., Ltd. (Wuhan, China); and A172 was purchased from the National Collection of Authenticated Cell Cultures (Shanghai, China), where it was characterized through mycoplasma detection. The genomic aberrations of these glioma cell lines were comprehensively analyzed (Melendez et al., 2011). The cells were cultured in high-glucose DMEM supplemented with 10% fetal bovine serum (FBS) and 1% penicillin/streptomycin at 37°C, and 5% CO_2_.

### Cell Viability Assay

The cell viability assay was evaluated in triplicate by using Cell Counting Kit-8 (CCK-8, #C6005, NCM Biotech) according to the manufacturer’s protocol. In brief, glioma cells (1 × 10^4^ cells/well) were cultured in 96-well plates for 24 h and then treated with TIZ at 0, 0.05, 0.1, 1, and 10 µM in various wells. After incubation for 24, 48, or 72 h, 10 µl CCK-8 was added to each well and incubated for 1 h at 37°C. The OD_450_ was measured using a microplate reader (Infinite F50, TECAN). Each assay was repeated three times.

### Colony Formation Assay

Glioma cells (500 cells/well) were seeded in a 6-well plate and treated with various concentrations of TIZ for 2 weeks. Next, the cells were stained with a 0.5% crystal violet solution for 15 min after washing with PBS and fixing with methanol. After washing with PBS thrice, the cells were dried in the air. The colonies with more than 50 cells were counted under a microscope. The colony-forming efficiency was calculated based on the following formula: colony-forming efficiency = (the number of colonies forming units/the number of inoculated cells) × 100%.

### ROS Staining Assay and Flow Cytometric Analysis

U87MG cells were treated with TIZ at the indicated concentrations, negative control included pretreatment with N-acetyl-L-cysteine (NAC, 5 mM) for 2 h before 10 µM TIZ treatment for 24 h, and then fixed with 4% paraformaldehyde for 10 min after washing with PBS. Cells were firstly washed with carrier buffer (1% BSA, 0.3% Triton X-100, and 1% goat serum in PBS) and then treated with 10 µM DCFH-DA that was diluted in PBS for 20 min at 37°C. Finally, a carrier buffer was used to wash the cells three times, and a confocal microscope was employed to obtain the images. For flow cytometric analysis, U87MG cells were resuspended in 10 µM DCFH-DA for 20 min at 37°C after treatment with TIZ for 24 h and then subjected to the flow cytometry to analyze the degree of fluorescence.

### Transfection of siRNA

The target sequence (5′-3′) of human siRNA-CDK1 (NM_001786) is ACT​TCG​TCA​TCC​AAA​TAT​A; sense: 5′-ACU​UCG​UCA​UCC​AAA​UAU​A dTdT-3′; antisense, 3′-dTdT UGA​AGC​AGU​AGG​UUU​AUA​U-5′. The negative control siRNA (F: 5′-UUC​UCC​GAA​CGU​GUC​ACG​UTT-3′; R: 5′-ACG​UGA​CAC​GUU​CGG​AGA​ATT-3) (Xiao et al., 2009). These siRNAs were chemically synthesized by GenePharma Co. Ltd. (Shanghai, China). U87MG cells were transfected with 20 nM siRNA using the Lipofectamine 3000 (#L3000075, Invitrogen) according to the manufacturer’s protocol with Opti-MEM (#31985070, Gibco) as a transaction solution. The treated cells were incubated for 48 h and then harvested for further experiments.

### Western Blotting

Cells or brain tissues were sonicated and lysed with a RIPA buffer and insoluble pellets were removed by centrifugation at 15,000 × *g* for 15 min at 4°C. Protein concentration was measured with a BCA kit. Equal amounts of protein (20–40 µg) were loaded for blotting with the corresponding antibodies. The quantitation of the western blot results was based on three independent experiments using ImageJ with the vehicle group as a baseline for comparison.

### Apoptosis Assay

U87MG cells were treated with different doses of TIZ for 48 h, negative control included pretreatment with NAC (5 mM) for 2 h before 10 µM TIZ treatment, and then harvested, and washed with PBS. Cells were stained with an Annexin V-PE/7-AAD Apoptosis Detection Kit (Solarbio, Beijing, China) for 20 min at room temperature followed by flow cytometry. The data were analyzed with Flow Jo software (Tristar, CA, USA).

### Cell Cycle Analysis

U87MG cells at a density of 4 × 10^5^ cells/well were exposed to TIZ for 24 h, fixed in 75% ethanol at 4°C for 24 h, and stained with a PI/RNase staining buffer for 30 min for flow cytometric analysis.

### Xenograft Animal Model Experiments

Female 6-week-old BALB/C nude mice were provided by Hunan SJA Laboratory Animal Co. Ltd. Mice were housed, maintained, and treated at the Central Laboratory of the Second Xiangya Hospital with SPF feeding conditions. For the subcutaneous tumor model, 100 µl PBS containing 1 × 10^7^ U87MG cells was inoculated subcutaneously into the right side of the axilla of each mouse. Tumor growth was calculated every 2 days according to the formula of TV (mm^3^) = (width)^2^ × (length)/2. When the tumor volume reached approximately 100 mm^3^, the mice were randomly divided into three groups (*n* = 6). For the orthotopic intracranial tumor model, mice were placed in a stereotaxic instrument, then 1 × 10^7^ U87MG-luc cells (10 µl) were performed stereotaxically at coordinates AP −2.0 mm and ML +0.7 mm relative to bregma and DV −3.0 mm from the dural surface. The needle was retained on site for 5 min before it was removed slowly. The mice were placed on a heating pad until they began to recover from the surgery. After surgery for 7 days, the mice were examined with an *in vivo* imaging system (IVIS) to validate tumor formation and then were randomly divided into three groups (*n* = 8). The mice in the above two models were administered TIZ (i.p., 5 mg/kg and 15 mg/kg) or a control agent three times per week for 3 weeks. After the drug treatment, mice in the intracranial model were euthanized and analyzed by MRI (Liu et al., 2019), after which samples were collected for subsequent studies. Bodyweight changes in mice were monitored throughout dosing and blood was collected to assess possible side effects of TIZ. The animal experiments were carried out following the Guiding Principles of the Animal Ethics Committee of the Second Xiangya Hospital of Central South University.

### Immunohistochemistry and Hematoxylin-Eosin Staining

Formalin-fixed samples were embedded in paraffin and sliced into 5 µm-thick sections. The sections were stained with a standard H&E staining protocol (Fischer et al., 2008). For IHC staining, the sections were treated with 0.3% hydrogen peroxide for 10 min followed by incubation with anti-Ki67 and anti-p-CDK1 Thr161 at 4°C overnight. After a brief wash, the brain tissue slices were incubated with a biotinylated secondary antibody and visualized by using a DAB substrate Kit for 10 min. The slices were then counterstained with hematoxylin, and pictures were captured on a microscope (BX51TF, Olympus, Tokyo, Japan). For quantification of positive cells and image analysis, set a proper threshold for the binarization of the selected color image by ImageJ software. The average optical density (AOD) was calculated according to a reported method (Chlipala et al., 2020). The conditions of the analysis were blinded to the investigator.

### Determination of TIZ in Glioma

The concentration of TIZ in glioma was determined by referring to the method previously reported (Guo et al., 2020). In brief, glioma tissue with 10 µl standard stock solution of TIZ and topiramate (internal standard, 5 µg/ml) was homogenized in cold acetonitrile (1:5, m/v) in a Tissuelyser ball mill (Servicebio, Wuhan, China), then sonicated for 5 min and centrifuged for 15 min at 4°C. 200 µl of supernatant of samples was removed and dried utilizing flowing nitrogen. Residual samples were reconstituted in 100 µl of acetonitrile containing 10% *N, N*-dimethylformamide (DMF) and analyzed on an HPLC system (Shimadzu, Kyoto, Japan) connected to an AB Sciex 4000 QTRAP mass spectrometer (AB Sciex, MA, USA). The compounds were separated on a Cosmosil 5C_18_-MS-II column (4.6 × 150 mm, 5 µm) with the temperature of the column oven was 30°C. The mobile phase was composed of acetonitrile and deionized water mixed with 10 mM ammonium formate (pH 3.0) according to a gradient volume ratio (60:40 to 80:20 to 60:40) with a flow rate of 0.8 ml/min. TIZ was detected in the positive mode by comparing the retention time and *m/z* ratio of the TIZ standard. Final concentrations of TIZ were adjusted for the weight of each glioma tissue.

### Predictive Target Collection, Gene Ontology, Pathway Enrichment, and Bioinformatic Analyses

The potential target collection of TIZ was performed using Seaware software, the Swiss Target Prediction database (http://www.swisstargetprediction.ch) and the Bioinformatics & Evolutionary Genomics database (http://bioinformatics.psb.ugent.be/webtools/Venn). The GeneCards database (https://www.genecards.org) and CTD database (http://ctdbase.org) were used to obtain the currently reported genes related to glioma. The DAVID database (https://david.ncifcrf.gov, Version 6.8) was used to perform gene ontology and pathway enrichment analyses. GO analysis annotated and classified genes according to biological process (BP), molecular function (MF), and cellular location (CC). The enriched biological pathways were determined with the KEGG datasets. Bioinformatic data analysis was obtained from the TCGA data portal (https://cancergenome.nih.gov/dataportal/data/about) and GEPIA (http://gepia.cancer-pku.cn).

### Molecular Docking

MOE2019 software was used to perform molecular docking. CDK1 (ID 6GU6) (Wood et al., 2019a), CDK2 (ID 6Q4G) (Wood et al., 2019b) and CDK4 (ID 4GCJ) (Schonbrunn et al., 2013) were obtained from Protein Data Bank. The 2D structure of TIZ was drawn in ChemDraw and converted to a 3D structure in MOE through energy minimization. Before docking, the force field of AMBER10: EHT and the implicit solvation model of Reaction Field (R-field) were applied to model molecular mechanics minimizations. MOE-Dock was used for molecular docking simulations of the TIZ with proteins. The “induced fit” protocol was selected, in which the side chains of the binding site in the receptor were allowed to move according to ligand conformations, and a constraint was applied on their positions. The weight used for tethering side-chain atoms to their original positions was 10. Firstly, all docked poses were ranked by the London dG scoring function, then force field refinement was applied to the top 30 poses followed by a rescoring of the GBVI/WSA dG scoring function. The conformation with the lowest binding free energy was finally identified as the best probable binding mode.

### Statistical Analysis

All data are presented as the mean ± SD from three or more independent experiments. Histological data were analyzed using either Student’s *t*-test or one-way ANOVA with Tukey’s multiple-comparisons test. The threshold for significance for all experiments was set *p < 0.05, and smaller *p* values are represented as **p < 0.01 and ^#^ *p* < 0.001.

## Results

### TIZ Inhibits Glioma Cell Proliferation and Induces ROS Damage

**FIGURE 1:**
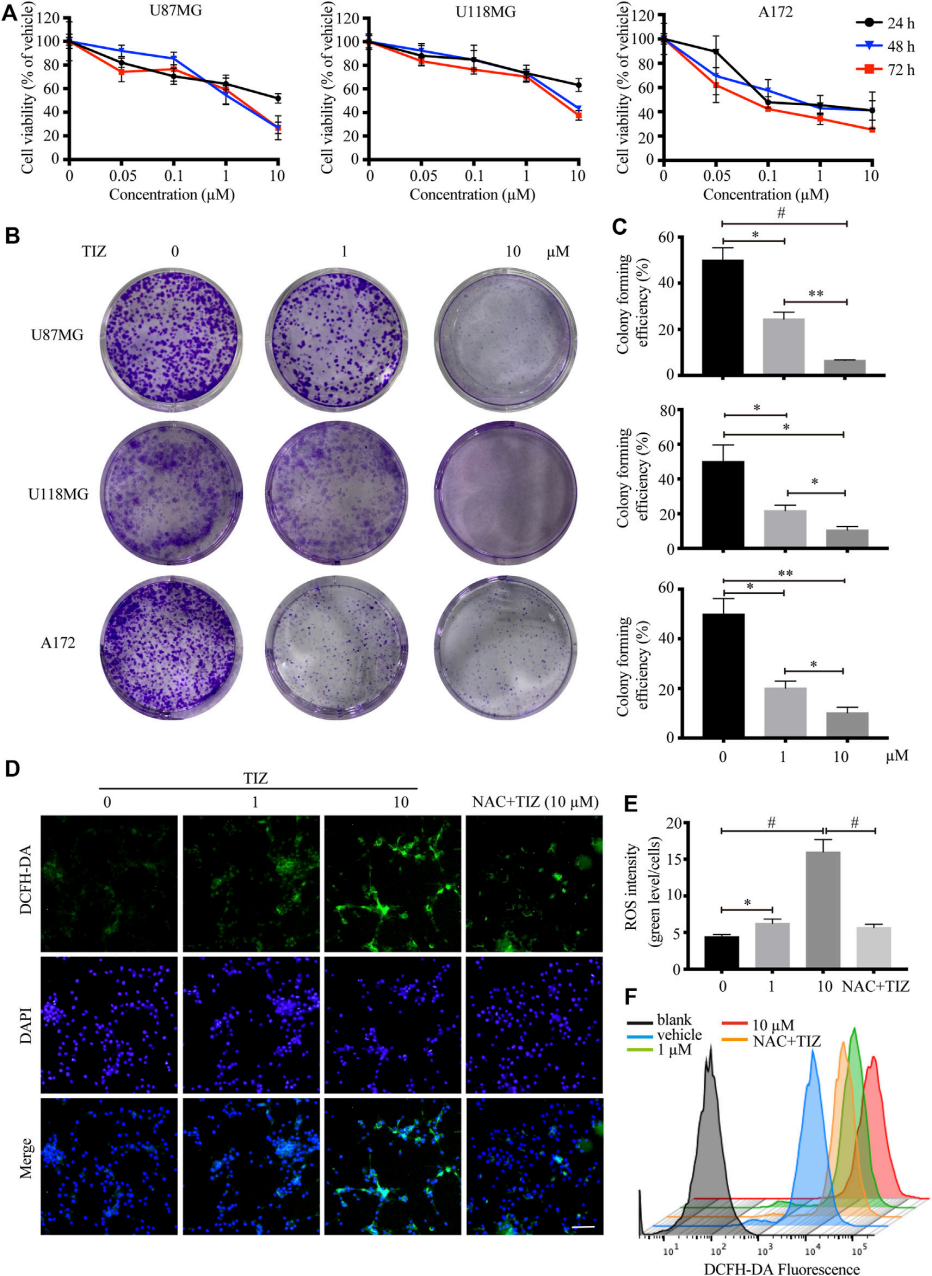
TIZ inhibits GBM cell growth and upregulates ROS levels *in vitro*. **(A)** The cell viability of U87MG, U118 MG, and A172 cells was determined by CCK-8 assays after 24, 48, and 72 h of TIZ treatment. **(B)** Representative images of U87MG colony, U118 MG colony, and A172 colony after the treatment with different concentrations of TIZ. **(C)** Quantification of colony area in colony formation assays (*n* = 3). **(D)** Representative images of ROS levels in various U87MG cells after the treatment with the indicated concentrations of TIZ for 24 h, NAC (5 mM) was pretreated for 2 h before TIZ treatment. ROS-positive cells were detected by the indicator dye DCFH-DA. Scale bar = 100 µm. **(E)** Quantification of ROS intensities using the ratio of fluorescent intensity and cell numbers (*n* = 5). **(F)** Flow cytometric measurements of the average fluorescence intensity of U87MG cells. The experiments were repeated three times independently. Data are presented as the mean ± SD (*p < 0.05, **p < 0.01, ^#^ *p* < 0.001).

To investigate the role of TIZ in the proliferation of glioma cells, CCK-8 assays were carried out to determine cell viability. Three human glioma cells lines, U87MG, U118MG, and A172, were treated with different concentrations of TIZ ranging from 0.01 to 10 µM for 24, 48, and 72 h. The results demonstrated that TIZ dose-dependently inhibited cell proliferation regardless of the number of treatment days (Figure 1A). The 48 h IC_50_ of TIZ for U87MG cells was 1.10 µM, that for U118MG cells was 2.31 µM, and that for A172 cells was 0.73 µM. Meanwhile, colony formation assays were performed to examine the effect of TIZ on the formation of glioma colonies. The results showed that TIZ significantly decreased the colony formation of all three cell lines, especially at a TIZ concentration of 10 µM (Figure 1B). Quantitative analysis of the colony-forming efficiency mirrored these discoveries (Figure 1C). Furthermore, to explore whether the ROS levels are correlated with the antiglioma effect of TIZ, we examined ROS production using the fluorescent probe 2,7-dichlorodihydrofluorescein diacetate (DCFH-DA) (Zhao et al., 2021). An increase in ROS production was observed in U87MG cells after TIZ treatment compared with the vehicle group, significantly diminished by ROS scavenger NAC (Figures 1D,E). In particular, the nuclear ROS levels were augmented sharply in the 10 µM TIZ group, which suggested that damage to biological behavior in the nucleus might be involved in the antiglioma mechanism of TIZ. Fluorescence intensity analysis by flow cytometry confirmed the conclusion obtained by the microscopy (Figure 1F). Therefore, these data suggested that TIZ suppressed the viability and proliferation of human glioma cells, and the intranuclear activities might be disrupted in this process.

### Cyclin-Dependent Kinases Are Potential Targets of TIZ Against Glioma

**FIGURE 2:**
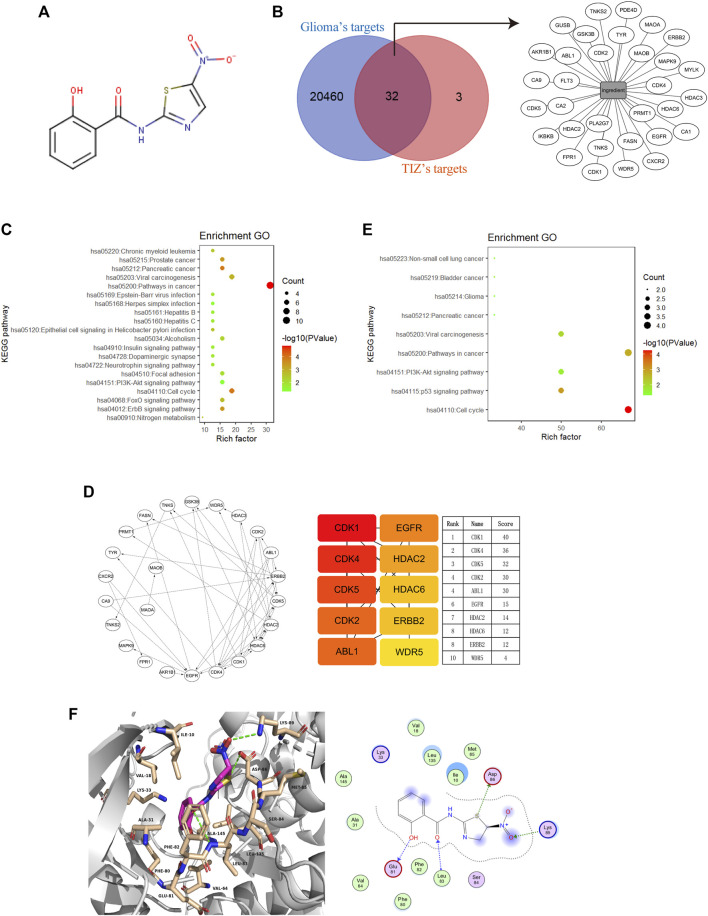
Cyclin-dependent kinases are potential targets of TIZ against glioma. **(A)** The structure of TIZ. **(B)** Venn diagram of the shared targets and the TIZ-target network. The related targets of GBM and TIZ were obtained from GeneCards and SEAware, respectively. **(C)** KEGG pathway enrichment analysis of the top 20 target genes. **(D)** Schematic diagram of target network connections and the top 10 targets and their scores. NetworkAnalyzer was employed to analyze the node’s degree and related parameters. The color of nodes changed from orange to red, which indicates that the affinity degree gradually increases from weak to strong. **(E)** KEGG pathway enrichment analysis of targets whose score was greater than 15. **(F)** Docking diagram of CDK1 and TIZ. Key interactions include hydrogen bonds formed by multiple atoms of TIZ with ASP86, Lys89, Glu81, and Leu83.

To explore the potential targets of TIZ in GBM, we performed target-binding predictions using SEAware software and Swiss Target Prediction based on the structure of TIZ (Figure 2A) and obtained a total of 35 corresponding potential target genes (Supplementary Table S1). A total of 20492 genes related to glioma were also collected in the GeneCards and CTD databases (data not shown).

Comparing the data of the two groups by using bioinformatics & evolutionary genomics, 32 promising antiglioma targets of TIZ were encapsulated, as shown in Figure 2B. The results from the Gene ontology enrichment analyses of the top 20 target genes showed that the genes enriched in the biological processes (BP) category involved protein autophosphorylation, cell proliferation, and apoptotic processing (Supplementary Figure S1A). In the molecular function (MF) category, the target genes were mainly involved in protein binding (Supplementary Figure S1B). In the cell components (CC) category, both the cytosol and nucleus contained these targets (Supplementary Figure S1C). KEGG pathway enrichment analysis showed an enhanced cell cycle of pathways in cancer (Figure 2C). A detailed *p* value analysis is provided in Supplementary Table S2.

To further anchor the potential targets, a top10 gene network was constructed using the cytoHubba module in Cytoscape, and the node’s degree and related parameters were analyzed by Network Analyzer (Figure 2D). Cyclin-dependent kinases (CDKs) 1, 4, 5, and 2 ranked at the top of the score, indicating that TIZ may act directly on these proteins to inhibit the cell cycle of glioma cells. KEGG pathway enrichment analysis of targets with scores greater than 15 mirrored the conclusion that the cell cycle was probably involved in the pharmacological mechanism of TIZ (Figure 2E). A detailed *p* value is provided in Supplementary Table S3.

To gain insight into the role of CDKs in human GBM, we analyzed a dataset from The Cancer Genome Atlas (TCGA) featuring expression and the corresponding clinical data of the selected GBM patient samples. Notably, when compared with the normal brain tissues, in addition to CDK5, the mRNA expression of CDK1, CDK2, and CDK4 was highly expressed in GBM patient samples (Supplementary Figure S1D). Next, we performed docking to elucidate the binding mode of TIZ with CDK1 (PDB ID: 6GU6). From the generated docking model, we found that TIZ was located in the active pocket of CDK1 with a calculated binding free energy of −36 kcal/mol by the GBVI/WSA dG scoring function, and formed multiple hydrogen bonds with ASP86, Lys89, Glu81, and Leu83 (Figure 2F). In parallel, the binding free energy of TIZ with CDK2 (PDB ID: 6Q4G) and CDK4 (PDB ID: 4GCJ) was calculated to be −25 kcal/mol and −19 kcal/mol, respectively (Supplementary Figures S1E,F). Although all calculated binding forces of TIZ with these three target proteins were smaller than those of TIZ with the ligand dinaciclib in the 6GU6 crystal (Supplementary Figure S1G, −41 kcal/mol), the docking results showed that TIZ has an affinity for CDKs, especially CDK1. Thus, these findings suggested that CDKs are potential targets of TIZ against GBM.

### TIZ Arrests the Cell Cycle at G2/M Phase and Induces Apoptosis of GMB by Inhibiting the Activities of CDK1

**FIGURE 3:**
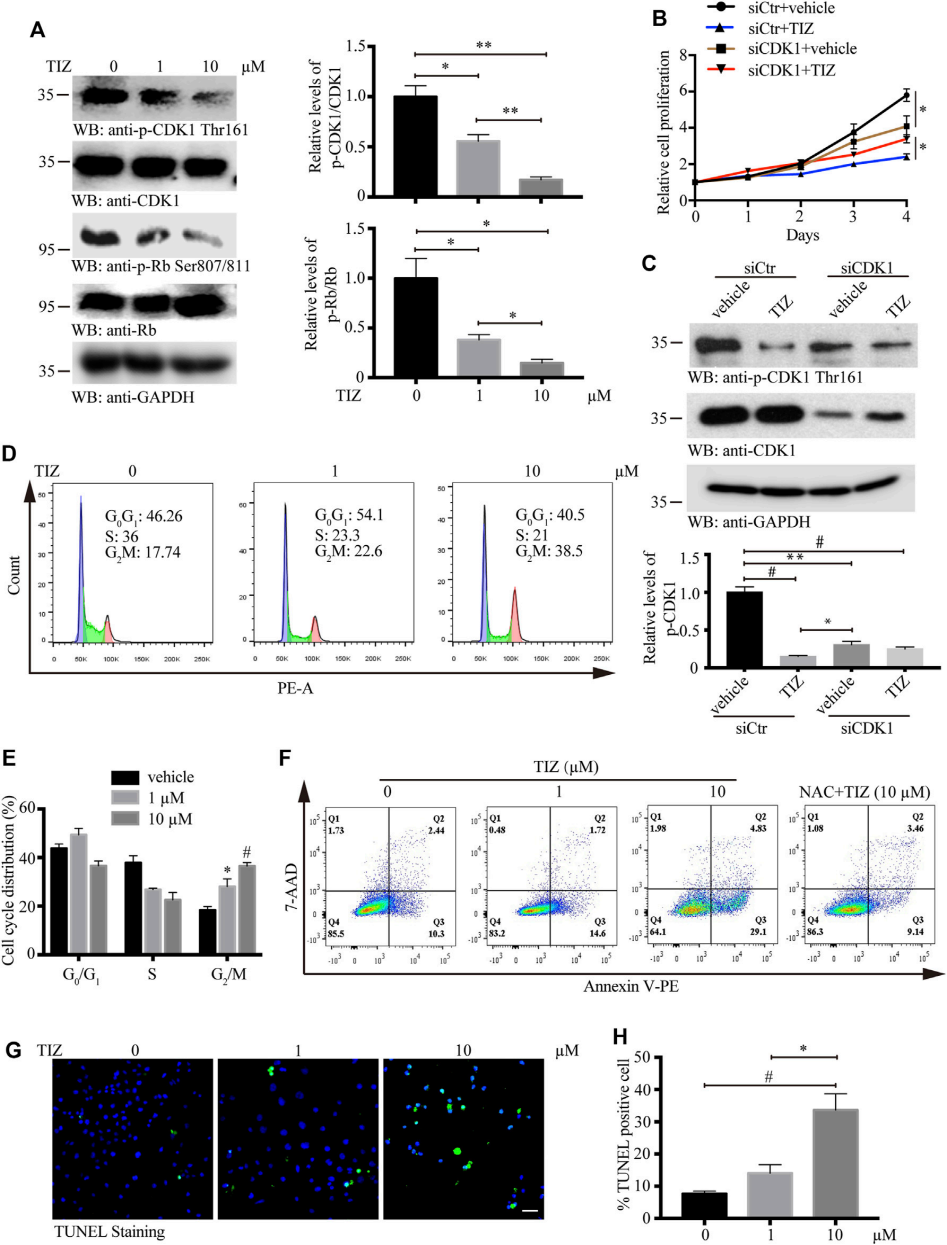
TIZ arrests the cell cycle at the G2/M phase and induces apoptosis of GMB by inhibiting the activities of CDK1. **(A)** TIZ inhibits the activity of CDKs. U87MG cells were treated for 48 h with the indicated concentration of TIZ. Cell lysates were immunoblotted with anti-phospho CDK1 Thr161, a marker of activated CDK1, and anti-phospho Rb Ser807/811, a marker of cellular CDK activity. Right, Quantification of the relative levels of p-CDK1 Thr161 and p-Rb Ser807/811 (*n* = 3). **(B)** CCK-8 assay of U87MG cells treated with TIZ (10 µM) or siCDK1 or their combination for up to 4 days. **(C)** Western blot analysis of U87MG cells treated with TIZ (10 µM), siCDK1, or their combination for 48 h. Down, quantification of the relative levels of CDK1 (*n* = 3). **(D)** Flow cytometric analysis of cycle distribution showed that TIZ arrested the cell cycle of U87MG cells at the G2/M phase. **(E)** Quantification of cycle distribution confirmed G2/M phase arrest (*n* = 3). **(F)** Flow cytometric analysis of apoptosis in cells double-stained with Annexin V-PE and 7-AAD. **(G)** TUNEL staining of U87MG cells after the treatment with the indicated concentration of TIZ for 48 h. Scale bar = 50 µm. **(H)** Quantification of TUNEL-positive cells (*n* = 5). Blot data are representative of three independent experiments. Data are presented as the mean ± SD (*p < 0.05, **p < 0.01, ^#^ *p* < 0.001).

During the embryonic breeding period, CDK1 binds to all cyclins, resulting in the phosphorylation of the retinoblastoma protein (pRb), and embryos fail to develop to the morula and blastocyst stages in the absence of CDK1. CDK1 was identified as the only essential cell cycle CDK and could execute all the events that are required to drive cell division (Santamaría et al., 2007). To validate whether CDK1 is the core target of TIZ, U87MG cells were treated with different concentrations of TIZ (1 and 10 µM) for 24 h, and cell lysates were immunoblotted with anti-CDK1 Thr161, a marker of activated CDK1, and anti-Rb Ser807/811, a marker of cellular CDK activity. As expected, the phosphorylation levels of these two proteins were significantly inhibited by TIZ (Figure 3A). To examine whether CDK1 is a direct molecular target of TIZ responsible for its antiproliferative effect, we treated U87MG cells with either siRNA control or CDK1-specific siRNA, followed by treatment with vehicle or TIZ for 4 days and monitoring of cell proliferation. Notably, the depletion of CDK1 reduced cell proliferation but compared with cells transfected with control siRNA, depletion of CDK1 attenuated the inhibitory effect of TIZ, which indicated that the inhibition of CDK1 is involved in the antiproliferative effect of TIZ (Figure 3B). Western blotting confirmed the successful knockdown of CDK1 and reflected the levels of p-CDK1 Thr161 (Figure 3C). Together, these data showed that CDK1 is the main cellular target of TIZ.

Inspired by the target determination process, flow cytometric analysis by propidium iodide was performed to investigate whether the observed TIZ effect on glioma cell viability was due to cell cycle arrest. As shown in Figure 3D, the percentage of cells in the S phase was reduced from 36% to approximately 22%, but notably, the cell rates in G2/M phase were augmented from 17.7% to 38.5% after the TIZ treatment at 1 and 10 μM, respectively. Quantitative analysis of cell cycle distribution mirrored these discoveries (Figure 3E). Based on cell cycle analysis, we quantitatively analyzed the apoptotic effect of TIZ using apoptosis-Annexin V/PE and 7AAD double-fluorescence staining by flow cytometry. The results indicated that the 48 h TIZ treatment significantly induced concentration-dependent apoptosis in U87MG cells. As shown in Figure 3F, the early apoptosis rate increased from 14.6% to 29.1% when the concentration increased from 1 to 10 μM, whereas the proportion of apoptotic cells was only 10.3% and 9.14% in the vehicle control and NAC pretreatment, respectively. We used another method to further confirm the proapoptotic effect of TIZ by a TUNEL staining. The results showed that TIZ also induced late apoptosis due to DNA damage in U87MG cells (Figures 3G,H). Therefore, our results indicated that TIZ arrests the cell cycle at the G2/M phase to induce apoptosis of GMB by inhibiting the activities of CDK1.

### TIZ Inhibits GBM Progression in Subcutaneous Xenograft in Nude Mice and Exhibits Preliminary Safety

**FIGURE 4:**
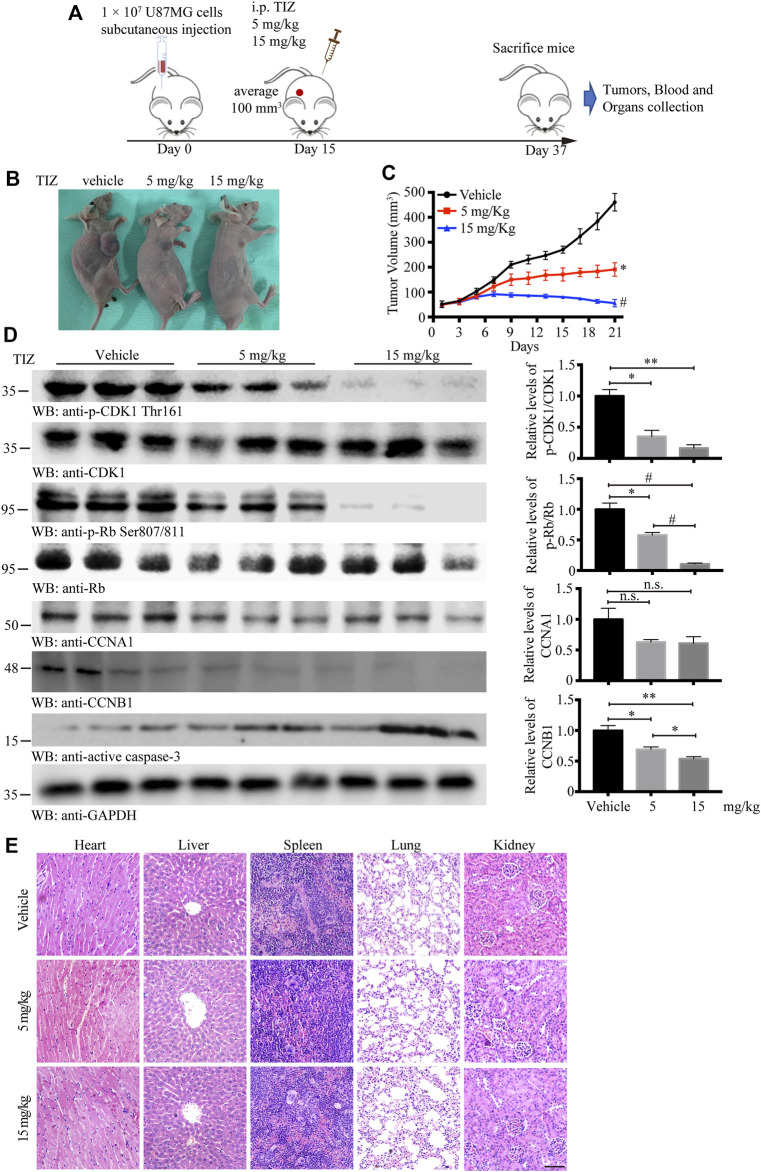
TIZ inhibits GBM growth in a subcutaneous xenograft nude mouse model. **(A)** Schematic of xenograft mouse model establishment and TIZ administration. **(B)** Representative images of GBM growth after different dosages of TIZ administration. **(C)** TIZ inhibited GBM growth as measured by tumor volume (*n* = 6). **(D)** Western blot analysis of tumor lysates with various indicated antibodies. Quantifications of relative protein levels are shown in the right panel (*n* = 3). Blot data are representative of three independent experiments. **(E)** TIZ administration displayed no detectable toxicity. Histological analysis of H&E-stained tissue sections of representative mice in the TIZ or vehicle-treated group. Scale bar, 100 µm. Data are presented as the mean ± SD (n.s. means no significance, *p < 0.05, **p < 0.01, ^#^ *p* < 0.001).

To evaluate the antiglioma activity and preliminary safety of TIZ *in vivo*, a subcutaneous xenograft model was established by inoculating U87MG nude mice and intraperitoneally treating the mice with 5 or 15 mg/kg of TIZ or vehicle three times per week for 3 weeks after the tumor volume reached approximately 100 mm^3^ (Figure 4A). Representative photographs of mice from each group were taken at the endpoint (Figure 4B). Consistent with the gross observations, TIZ significantly reduced tumor volumes compared to the rapidly growing tumors in the vehicle group (Figure 4C). Next, we checked the related protein expression levels in the tumor tissue samples. Consistent with the *in vitro* results, the phosphorylation levels of CDK1 Thr161 and Rb Ser807/811 were inhibited, and active caspase 3 was increased by TIZ in a dose-dependent manner. CCNB1 (Cyclin B1), a protein that can form a complex with CDK1 in the G2/M phase, was accordingly reduced. However, the expression levels of CCNA1 (Cyclin A1) showed no obvious change between the experimental groups (Figure 4D). H&E staining demonstrated no obvious lesions in the heart, liver, spleen, lung, or kidney (Figure 4E). Together with the analysis results of RBCs and WBCs from the whole blood, as well as ALT, AST, and CREA from serum, we found that the side effects of TIZ were modest (Supplementary Figure S2), which suggested that TIZ does not have detectable systemic toxicity.

### TIZ Suppresses the Intracranial Growth of GBM and Prolongs Mouse Survival

**FIGURE 5:**
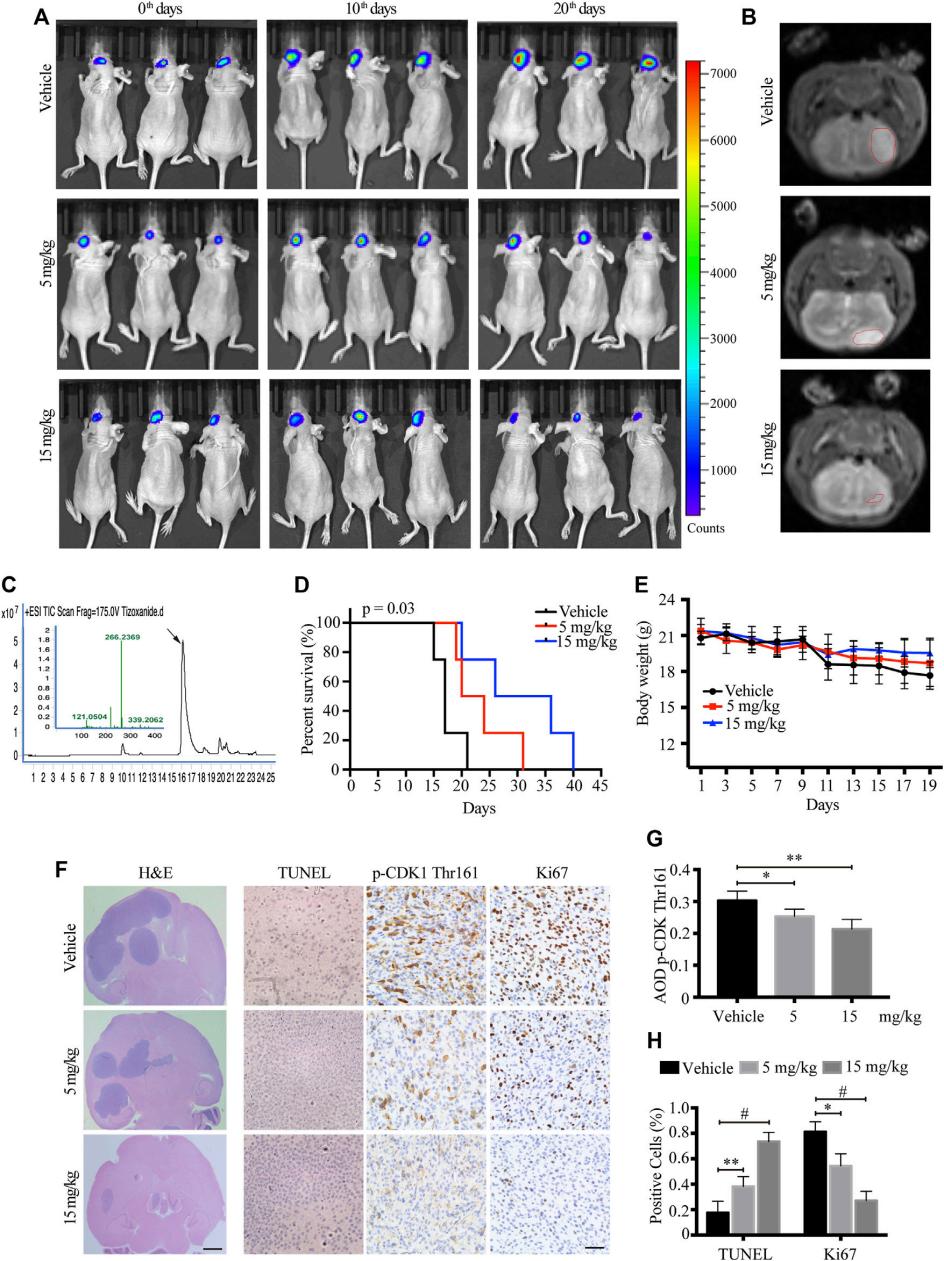
TIZ suppresses the intracranial growth of GBM and elongates mouse survival. **(A)** IVIS analysis of fluorescence signal intensity in the brain at different stages of TIZ administration (*n* = 12 mice per group). **(B)** Representative images of brain tumor volume scan by the small animal MRI system. **(C)** Representative HPLC-MS chromatograms of the concentration analysis of TIZ in the glioma homogenate samples. **(D)** The survival curves of mice bearing glioma in the brain. **(E)** Bodyweight curves of nude mice during TIZ treatment. **(F)** H&E, TUNEL, and p-CDK1 Thr161 and Ki67 IHC staining of tumor slices in various groups. Scale bar = 1 mm (left) and 50 µm (right). Quantification of IHC staining of p-CDK1 Thr161 **(G)** and positive TUNEL and Ki67 staining **(H)** (*n* = 5).

To monitor the therapeutic effect of TIZ on GBM in greater detail, we further used a murine intracranial xenograft model *in situ* by stereotaxically injecting U87MG-luc cells into the brain. After confirming tumor formation by IVIS, each group of nude mice was administered TIZ with the same schedule of subcutaneous mode. Representative images monitored by IVIS were captured on the 0th,10th, and 20th days and showed that TIZ attenuated the growth of tumors (Figure 5A). A week after drug treatment, mice were subjected to magnetic resonance imaging (MRI) and mirrored the IVIS results (Figure 5B). To investigate the efficiency and blood-brain barrier (BBB) permeability of TIZ, we determined the concentration of TIZ in glioma after intraperitoneal injections of 5 and 15 mg/kg using HPLC-MS (Figure 5C). The results showed that TIZ can cross BBB and the average concentration reached 6.978 μg/kg and 20.084 μg/kg, respectively (Supplementary Table S4). The median survival compared by Kaplan-Meier analysis showed that TIZ at 15 mg/kg enhanced mouse survival (Figure 5D), and the body weight of mice in the drug group remained relatively stable compared with the weight loss in the vehicle group (Figure 5E). Furthermore, we harvested and sliced brains after sacrificing nude mice and performed histological staining (Figure 5F). Tumor size tracked by H&E staining of the entire brain slice confirmed that TIZ significantly suppressed the growth of GBM, especially at the dosage of 15 mg/kg (Figure 5F, left). As expected, the TUNEL assay showed extensive apoptosis in the TIZ-treated groups, and IHC staining for Ki67 indicated an almost 55% reduction in the number of proliferating cells in the 15 mg/kg TIZ group compared to the vehicle group, which was in alignment with the p-CDK1 Thr161 staining (Figure 5F right). Quantification of IHC staining confirmed these findings (Figures 5G,H). Thus, the data supported our previous observations that TIZ significantly blocks GBM growth by suppressing cell proliferation and inducing apoptosis by inhibiting the activity of CDK1 *in vivo*.

## Discussion

The deregulated proliferation and inhibition of apoptosis lie at the heart of all tumor development, which presents two definite directions for therapeutic intervention in all cancers. Thus, the capability to induce cell cycle arrest and promote apoptosis is a common criterion of potential chemotherapeutic agents (Liu et al., 2012). The augmentation of cell proliferation is largely due to tumor cells suffering defects that derail the cell cycle machinery. Such defects can target either mutation of upstream signaling cascades, such as mitogen protein tyrosine kinase (PTK) and G-protein signal transducers, or elements of the cell cycle itself that both eventually converge to trigger cell cycle deregulation (Malumbres and Carnero, 2003). In the present study, we identified TIZ as a novel inhibitor of the cell cycle and induced apoptosis in GBM. Although NTZ was first reported as an autophagy inhibitor and induced cell cycle arrest in GBM by upregulating ING1 (p33) expression (Wang et al., 2018), it is intrinsically linked with our finding that CDK1 is a target of TIZ. Because CDK1 and checkpoint kinase 1 (CHK1) can phosphorylate p33^ING1b^ at Ser-126 in non-stress and DNA damage conditions, respectively, which increases the stability of p33^ING1b^ (Garate et al., 2007). Thus, it can be speculated that when CDK1 activity is inhibited by TIZ, CHK1 replay predominantly phosphorylates p33^ING1b^ at Ser-126 followed by the upregulation of p33^ING1b^. Meanwhile, we identified CDK1 as a direct affinity target of TIZ and found that the antiglioma efficiency of TIZ was more robust than that of NTZ by referring to the previously reported data (Wang et al., 2018). NTZ has been reported to spontaneously hydrolyzes to TIZ once it dissolves in water and reaches equilibrium in 10 h (Qu et al., 2018), which could provide a plausible explanation for the antitumor advantages of TIZ.

In the process of finding a target for TIZ, we took a strategy based on network pharmacological disease target analysis together with computerized compound-target seeking technology, which is frequently used in the initial stage of innovative drug development (Gu et al., 2020). We obtained a series of candidate direct-binding proteins of TIZ, including CDK1, CDK4, CDK5, CDK2, and ABL1, which were ranked in the top five (Figure 2). In terms of CDKs, unlike unicellular organisms such as yeasts, only a single cyclin-dependent kinase, CDK1, is required to drive cell division (Michowski et al., 2020). Mammalian cells are traditionally believed to require the sequential activation of CDKs, such as CDK1, CDK2, CDK3, CDK4, and CDK6, to push interphase proceeding through mitosis (Malumbres and Barbacid, 2005). However, a report published in 2007 indicated that CDK1 is the only essential cell cycle CDK; in the absence of interphase CDKs, CDK1 can execute all the events that are required to drive cell division (Santamaría et al., 2007). Following this point of view, although the results of molecular docking supported the existence of affinity potential of TIZ with CDK1, CDK2, and CDK4, we only performed siRNA targeting CDK1 to verify the inhibitory effect of TIZ on the proliferation of gliomas. In addition, the CDK family comprises 21 phosphotransfer enzymes (CDK1-21) with diverse cellular functions, the development of CDK inhibitors with isozyme selectivity is technically challenging due to the catalytic pocket across the CDK enzyme family being highly conserved. Five cell-permeable energy transfer probes were tested to comprehensively profile CDK engagement in live cells (Wells et al., 2020). However, these probes are still on the way to commercialization, and we expect to get more accurate data about TIZ selectivity for CDK1-21 in the future and not just limited to CDK1, 2, 4, and 5 that are focused in our research. Tyrosine-protein kinase ABL1, targeting mitochondria in the response to oxidative stress and thereby mediating mitochondrial dysfunction and cell death, was disclosed as a target against *Entamoeba histolytica*, whereas NTZ is a classical drug for parasitic amoeba infection (Gilles and Hoffman, 2002; Sauvey et al., 2021). It will be interesting to verify the role of these TIZ direct binding proteins in different contexts.

An intriguing finding in the present study is that the downregulated expression of cyclin A1 is less desensitized than that of cyclin B1 after the GBM cells were treated with TIZ. Reviewing the entire mitotic process of mammalian cells, each meiotic, as well as mitotic G2 phase to M phase is initialized through the activation of a complex of enzymatic subunit CDK1 and the regulatory cyclin B1 (Masui, 2001). The stability and activity of CDK1/cyclin B1 are tightly regulated by the phosphorylation state of CDK1, both by activating phosphorylation at Thr161 and inhibitory phosphorylation at Thr14 and Tyr15 (Timofeev et al., 2010; Shi and Feng, 2021). When complex inactivation occurs, cyclin B1 dissociates from CDK1 and becomes polyubiquitinated and targeted by the proteasome, and cyclin B1 degradation is crucial for long-term CDK1/cyclin B1 inactivation, whereas cyclin A1 is expressed during meiosis and embryogenesis and plays a critical role in S phase progression as a complex with CDK2 (Kubiak et al., 2003; Hirayama et al., 2020). Because TIZ could directly inhibit CDK1 phosphorylation at Thr161 and inhibit the stability of CDK1/cyclin B1 to trigger cyclin B1 degradation, the downregulation of cyclin B1 was more sensitive than that of cyclin A1, which might be attributed to the mechanism by which TIZ caused G2/M arrest.

Reactive oxygen species (ROS) play an important role in controlling certain stages of the cell cycle. For instance, CDK1/cyclin B1 is inactivated by oxidation; conversely, the complex itself seems concomitant with ROS leakage at the G2/M transition (Wang et al., 2014; Chang et al., 2021). ROS levels have been observed to peak in mitosis, resulting in mitotic accumulation of oxidized protein cysteine residues (Patterson et al., 2019). The results of several studies suggested the possibility of ROS-mediated oxidative DNA damage in the cell cycle process (Mittal and Pandey, 2014; Xie et al., 2018). In this study, a dose-dependent augmentation in ROS levels, especially in the nucleus, was observed after TIZ treatment, together with NAC significantly attenuated the ROS levels and apoptosis induced by TIZ treatment (Figures 1D, 3F), implying damage to the DNA synthesis process. Mechanistically, our studies indicate that TIZ induces apoptosis of GBM mainly through ROS accumulation during G2/M arrest.

Drug repurposing refers to the reuse of clinical drugs to treat non-indication diseases. It has been a hot spot in drug development in recent years due to the compounds that have already been tested in humans and have demonstrated an acceptable level of safety and tolerability (Levin et al., 2020). NTZ is generally well tolerated, and adverse events have been mild and transient, and principally related to the gastrointestinal tract. Although in the subcutaneous xenograft mice, there was an unexplained rise in WBCs after TIZ administration (Supplementary Figure S2B), no significant adverse events have been noted in human trials as a new thiazolide antiparasitic agent (Fox and Saravolatz, 2005). NTZ is a FAD-approved drug that is used to treat infections by protozoa, helminths, anaerobic bacteria, microaerophilic bacteria, and viruses. The ADMET properties have been elucidated in the development process. The ADMET properties of TIZ could deeply refer to these of NTZ. Our results regarding the preliminary safety of TIZ as an antitumor candidate partially support the possibility of transitioning it into clinical research for a chemotherapy drug. Meanwhile, one situation we are facing is the continuation of the coronavirus disease 2019 (COVID-2019) pandemic, which has created panic and alarm across the globe. NTZ, as a first-in-class broad-spectrum antiviral agent, is widely reported to have the potential to treat COVID-2019 (Mahmoud et al., 2020; Rocco et al., 2020). To date, 10 clinical trial protocols focusing on evaluating the effects of NTZ in the treatment of COVID-19 have been registered at ClinicalTrials.gov, but preliminary results have not yet been reported (Martins-Filho et al., 2020).

## Conclusion

In summary, our results show that TIZ, a novel CDK1 inhibitor, has favorable efficacy in inhibiting GBM *in vitro* and *in vivo*. It promotes apoptosis in glioma cells by inducing G2/M cell cycle arrest by inhibiting the phosphorylation of CDK1 and the activity of the CDK1/cyclin B1 complex. Both *in vivo* subcutaneous and intracranial orthotopic xenograft models showed that TIZ could inhibit the growth of GBM and prolong the survival of nude mice. Moreover, TIZ has shown good safety profiles. Taken together, the results indicate that TIZ is a favorable small-molecule antiglioma drug candidate, and its further repurposing investigations are warranted.

## Data Availability

The original contributions presented in the study are included in the article/Supplementary Material, further inquiries can be directed to the corresponding author.

## Supplementary Material

Supplementary Figure S1 Bioinformatic analysis of the potential targets of TIZ and target-TIZ docking results. **(A)** Gene ontology (GO) biological process enrichment analysis of top20 target genes. **(B)** GO molecular function enrichment analysis of top20 target genes. **(C)** GO cellular component analysis of top20 target genes. **(D)** The mRNA expression of CDK1, CDK2, CDK4, and CDK5 were collected from The Cancer Genome Atlas (TCGA) and Gene Expression Profiling Interactive Analysis (GEPIA) databases. T, tumor; N, normal. **(E)**
Docking diagram of CDK4 and TIZ. Key interactions include hydrogen bonds formed by a hydrogen atom of TIZ with Asp86. **(G)** Docking result of CDK1 and itself ligand Dinaciclib. Dinaciclib is a novel potent small-molecule inhibitor of CDK1, CDK2, CDK5, and CDK9. It is being evaluated in clinical trials for various cancer indications.

Supplementary Figure S2 Analysis of blood biochemical parameters in nude mice after TIZ administration The results of RBC **(A)**, ALT **(C)**, AST **(D)**, and CREA **(E)** showed that Red blood cells and hepatic and renal function in mice have no obvious changes between the vehicle and TIZ -treated groups. The reason for the increase in white blood cells might be that an inflammatory response was stimulated during TIZ administration **(B)**. Data are presented as the mean ± SD (*n* = 5, **p* < 0.05, ***p* < 0.01, ^#^ *p* < 0.001).
