# Supplementary figures and images for: Tizoxanide Promotes Apoptosis in Glioblastoma by Inhibiting CDK1 Activity
Source: Front Pharmacol. 2022 May 25;13:895573. doi: 10.3389/fphar.2022.895573 (PMC9174573; doi:10.3389/fphar.2022.895573)

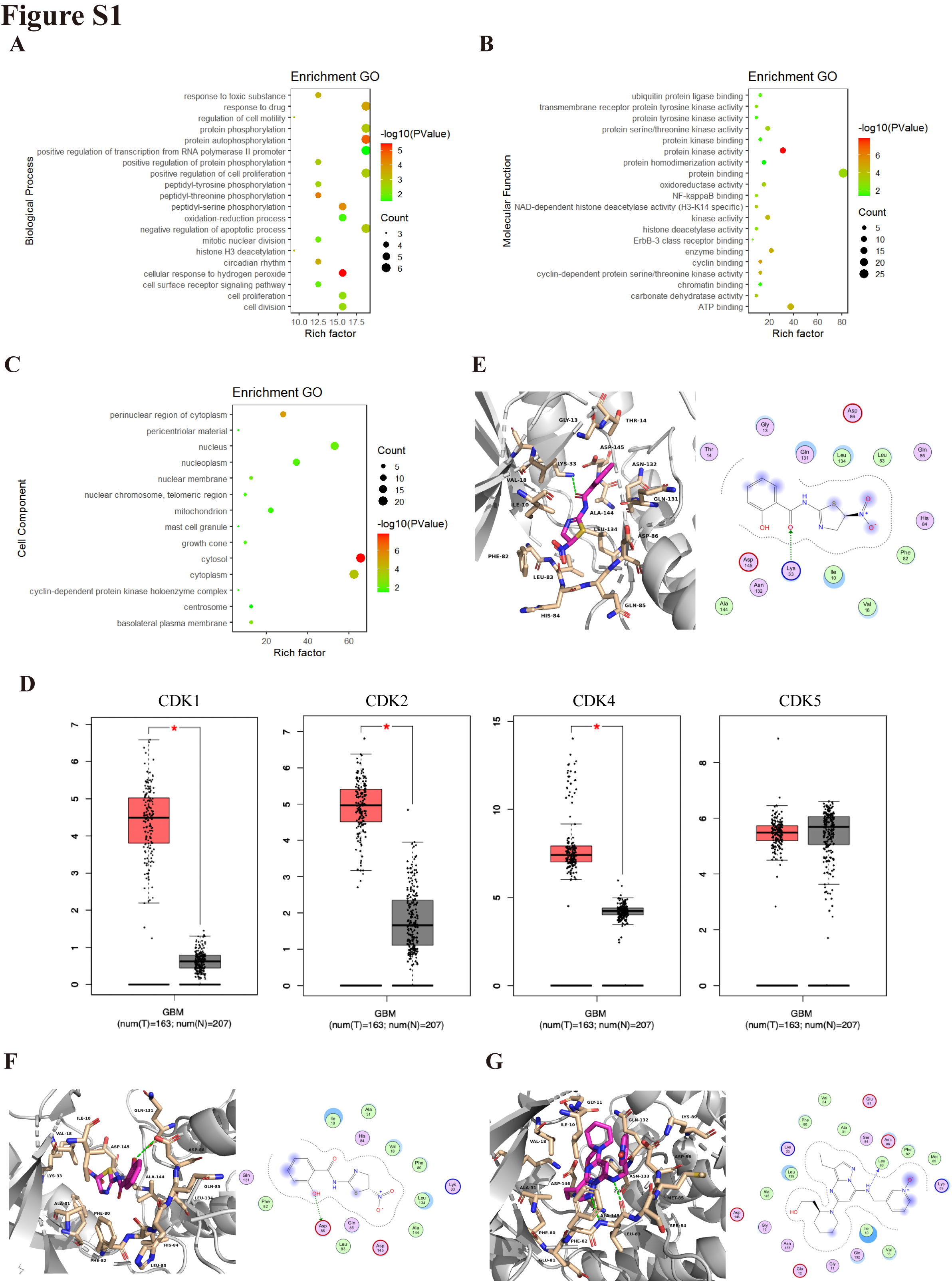

Supplement: Supplementary file 1 [file Image1.tiff]

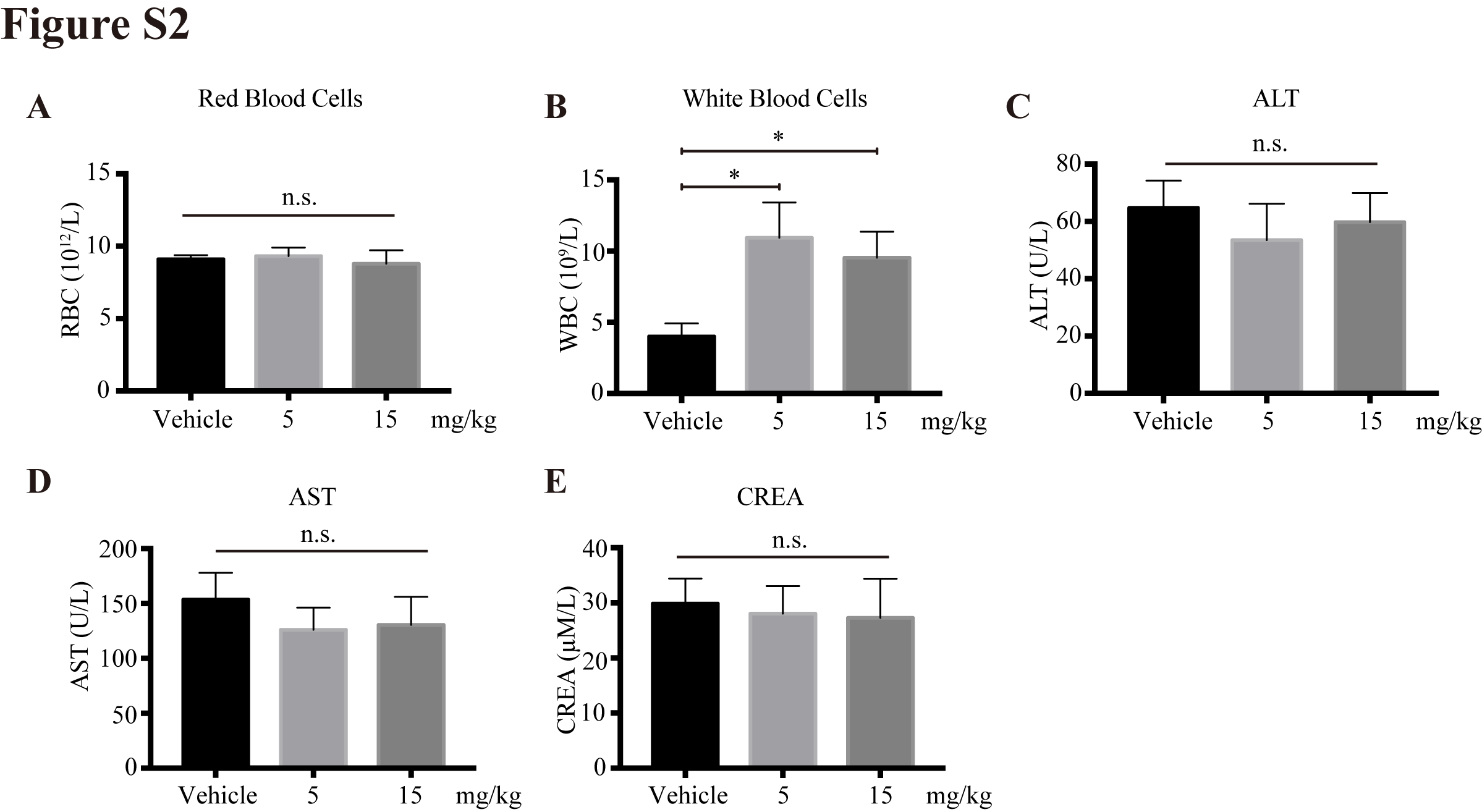

Supplement: Supplementary file 7 [file Image2.tiff]
