# Supplementary material for: Tizoxanide Promotes Apoptosis in Glioblastoma by Inhibiting CDK1 Activity
Source: Front Pharmacol. 2022 May 25;13:895573. doi: 10.3389/fphar.2022.895573 (PMC9174573; doi:10.3389/fphar.2022.895573)
Supplement: Supplementary file 2 [file Table1.docx]

**Table S1. 35 potential target genes of TIZ**

| **No.** | **Target gene** | **Description** | **Probability** |
| --- | --- | --- | --- |
| 1 | LOX15 | Arachidonate 15-lipoxygenase | 0.0535560755162 |
| 2 | ABL1 | Tyrosine-protein kinase ABL | 0.0535560755162 |
| 3 | AK1C2 | Aldo-keto reductase family 1 member C2 | 0.0535560755162 |
| 4 | FPR1 | Formyl peptide receptor 1 | 0.0535560755162 |
| 5 | CDK5 | Cyclin-dependent kinase 5 | 0.0535560755162 |
| 6 | WDR5 | WD repeat-containing protein 5 | 0.0535560755162 |
| 7 | PDE4D | Phosphodiesterase 4D | 0.0535560755162 |
| 8 | KCMA1 | Calcium-activated potassium channel subunit alpha-1 | 0.0535560755162 |
| 9 | AKR1B1 | Aldose reductase | 0.108018050868 |
| 10 | MAPK9 | c-Jun N-terminal kinase 2 | 0.0626219668353 |
| 11 | CA2 | Carbonic anhydrase II | 0.0535560755162 |
| 12 | CA1 | Carbonic anhydrase I | 0.0535560755162 |
| 13 | CA9 | Carbonic anhydrase IX | 0.0535560755162 |
| 14 | MAOA | Monoamine oxidase A | 0.0535560755162 |
| 15 | MAOB | Monoamine oxidase B | 0.0535560755162 |
| 16 | TNKS2 | Tankyrase-2 | 0.0535560755162 |
| 17 | TNKS | Tankyrase-1 | 0.0535560755162 |
| 18 | FASN | Fatty acid synthase | 0.0535560755162 |
| 19 | FLT3 | Tyrosine-protein kinase receptor FLT3 | 0.0535560755162 |
| 20 | CDK2 | Cyclin-dependent kinase 2 | 0.0535560755162 |
| 21 | CDK1 | Cyclin-dependent kinase 1 | 0.0535560755162 |
| 22 | CDK4 | Cyclin-dependent kinase 4 | 0.0535560755162 |
| 23 | PRMT1 | Protein-arginine N-methyltransferase 1 | 0.0535560755162 |
| 24 | IKBKB | Inhibitor of nuclear factor kappa B kinase beta subunit | 0.0535560755162 |
| 25 | GUSB | Beta-glucuronidase | 0.0535560755162 |
| 26 | ERBB2 | Receptor protein-tyrosine kinase erbB-2 | 0.0535560755162 |
| 27 | EGFR | Epidermal growth factor receptor erbB1 | 0.0535560755162 |
| 28 | TYR | Tyrosinase | 0.0535560755162 |
| 29 | CXCR2 | Interleukin-8 receptor B | 0.0535560755162 |
| 30 | HDAC3 | Histone deacetylase 3 | 0.0535560755162 |
| 31 | HDAC6 | Histone deacetylase 6 | 0.0535560755162 |
| 32 | HDAC2 | Histone deacetylase 2 | 0.0535560755162 |
| 33 | GSK3B | Glycogen synthase kinase-3 beta | 0.0535560755162 |
| 34 | MYLK | Myosin light chain kinase, smooth muscle | 0.0535560755162 |
| 35 | PLA2G7 | LDL-associated phospholipase A2 | 0.0535560755162 |
