# Supplementary material for: Tizoxanide Promotes Apoptosis in Glioblastoma by Inhibiting CDK1 Activity
Source: Front Pharmacol. 2022 May 25;13:895573. doi: 10.3389/fphar.2022.895573 (PMC9174573; doi:10.3389/fphar.2022.895573)
Supplement: Supplementary file 3 [file Presentation1.pdf]

Supplemental information

Tizoxanide promotes apoptosis in glioblastoma by inhibiting CDK1 activity

Si Huang[a,b], Jingxian Xiao[c], Junyong Wu[a,b], Jiayi Liu[d], Xueping Feng[e], Chengdong Yang[f], Daxiong Xiang[a,b], Shilin Luo[a,b,*]

[a] Department of Pharmacy, the Second Xiangya Hospital, Central South University, Changsha 410011, PR China

[b] Hunan Provincial Engineering Research Centre of Translational Medicine and Innovative Drug, Changsha 410011, PR China

[c] School of Medical Science, Hunan University of Medicine, Huaihua 418000, PR China

[d] Department of Radiology, the Second Xiangya Hospital, Central South University, Changsha, 410011, PR China

[e] Institute of Medical Sciences, Xiangya Hospital, Central South University, Changsha, 410078, PR China

[f] Department of Psychiatry, the Second Xiangya Hospital, Central South University, Changsha, 410011, PR China

# To whom all correspondence should be addressed (E-mail: Shilin_luo@csu.edu.cn)

Figure S1. Bioinformatic analysis of the potential targets of TIZ and target-TIZ docking results. (A) Gene ontology (GO) biological process enrichment analysis of top20 target genes. (B) GO molecular function enrichment analysis of top20 target genes. (C) GO cellular component analysis of top20 target genes. (D) The mRNA expression of CDK1, CDK2, CDK4, and CDK5 were collected from The Cancer Genome Atlas (TCGA) and Gene Expression Profiling Interactive Analysis (GEPIA) databases. T = tumor, N = normal. (E) Docking diagram of CDK2 and TIZ. Key interactions include hydrogen bonds formed by two oxygen atoms of TIZ with Lys33. (F) Docking diagram of CDK4 and TIZ. Key interactions include hydrogen bonds formed by a hydrogen atom of TIZ with Asp86. (G) Docking result of CDK1 and itself ligand Dinaciclib. Dinaciclib is a novel potent small-molecule inhibitor of CDK1, CDK2, CDK5, and CDK9. It is being evaluated in clinical trials for various cancer indications.

Figure S2. Analysis of blood biochemical parameters in nude mice after TIZ administration The results of RBC (A), ALT (C), AST (D), and CREA (E) showed that Red blood cells and hepatic and renal function in mice have no obvious changes between the vehicle and TIZ -treated groups. The reason for the increase in white blood cells might be that an inflammatory response was stimulated during TIZ administration (B). Data are presented as the mean ± SD (n = 5, *$P < 0.05$, **$P < 0.01$, #$P < 0.001$).
