# Supplementary material for: Tizoxanide Promotes Apoptosis in Glioblastoma by Inhibiting CDK1 Activity
Source: Front Pharmacol. 2022 May 25;13:895573. doi: 10.3389/fphar.2022.895573 (PMC9174573; doi:10.3389/fphar.2022.895573)
Supplement: Supplementary file 4 [file Table2.docx]

**Table S2. TOP10 KEGG enrichment pathway information of the anti-glioma**

| **Pathway** | **Count** | **P Value** |
| --- | --- | --- |
| Pathways in cancer | 10 | 1.55E-05 |
| Cell cycle | 6 | 1.24E-04 |
| Pancreatic cancer | 5 | 1.25E-04 |
| ErbB signaling pathway | 5 | 3.88E-04 |
| Prostate cancer | 5 | 4.05E-04 |
| Viral carcinogenesis | 6 | 0.001257194 |
| FoxO signaling pathway | 5 | 0.00196072 |
| Nitrogen metabolism | 3 | 0.002092459 |
| Epithelial cell signaling in Helicobacter pylori infection | 4 | 0.002430862 |
| Chronic myeloid leukemia | 4 | 0.002985493 |
