# Supplementary material for: Tizoxanide Promotes Apoptosis in Glioblastoma by Inhibiting CDK1 Activity
Source: Front Pharmacol. 2022 May 25;13:895573. doi: 10.3389/fphar.2022.895573 (PMC9174573; doi:10.3389/fphar.2022.895573)
Supplement: Supplementary file 5 [file Table3.docx]

**Table S3. KEGG enrichment pathway information based on target’s score**

| **Pathway** | **Count** | **P Value** |
| --- | --- | --- |
| Cell cycle | 4 | 5.57E-05 |
| p53 signaling pathway | 3 | 9.17E-04 |
| Pathways in cancer | 4 | 0.001697328 |
| Viral carcinogenesis | 3 | 0.00832845 |
| PI3K-Akt signaling pathway | 3 | 0.022673556 |
| Bladder cancer | 2 | 0.029456181 |
| Non-small cell lung cancer | 2 | 0.040057709 |
| Pancreatic cancer | 2 | 0.046374021 |
| Glioma | 2 | 0.046374021 |
| Melanoma | 2 | 0.050566391 |
