# Supplementary material for: Tizoxanide Promotes Apoptosis in Glioblastoma by Inhibiting CDK1 Activity
Source: Front Pharmacol. 2022 May 25;13:895573. doi: 10.3389/fphar.2022.895573 (PMC9174573; doi:10.3389/fphar.2022.895573)
Supplement: Supplementary file 6 [file Table4.docx]

**Table S4.** TIZ concentration in glioma after I.P. injection of 5 mg/kg or 15 mg/kg

| Group | Concentration(µg/kg) | | |
| --- | --- | --- | --- |
|  | 1 | 2 | 3 |
| vehicle | 0 | 0 | 0 |
| 5 mg/kg | 7.142 | 6.779 | 7.013 |
| 15 mg/kg | 20.493 | 19.119 | 20.641 |
